# Penile pseudomyogenic hemangioendothelioma: a rare case report with clinicopathologic features and multidisciplinary management

**DOI:** 10.3389/fmed.2026.1777602

**Published:** 2026-02-18

**Authors:** Cecilia Carrión, Paola Sánchez, Daniel Moreira, Marlon Arias-Intriago, Juan S. Izquierdo-Condoy

**Affiliations:** 1 Pathology Department, SOLCA Quito, Quito, Ecuador; 2 Departamento de Patología, Universidad Central del Ecuador, Quito, Ecuador; 3 One Health Research Group, Universidad de las Américas, Quito, Ecuador

**Keywords:** multidisciplinary management, penile tumor, perineural invasion, pseudomyogenic hemangioendothelioma, rare tumors

## Abstract

**Background:**

Pseudomyogenic hemangioendothelioma (PHE) is a rare vascular neoplasm of intermediate malignancy that predominantly affects the limbs and trunk of young adults. Genital localization, particularly penile involvement, is exceptionally uncommon and may lead to diagnostic challenges.

**Case presentation:**

A 29-year-old man presented with a painful, ulcerative lesion on the ventral prepuce that had been present for 5 years. He initially underwent circumcision, and histopathologic evaluation of the surgical specimen suggested an undifferentiated malignant neoplasm. Despite surgery, symptoms persisted. Three months later, magnetic resonance imaging (MRI) revealed a penile mass infiltrating the corpora cavernosa, corpus spongiosum, and glans. Subsequent histopathologic re-evaluation with immunohistochemistry demonstrated co-expression of cytokeratins and endothelial markers (CD31, ERG), establishing the diagnosis of PHE. The tumor showed perineural and intravascular invasion with positive surgical margins. Given the extent of local disease and the complex anatomic location, a multidisciplinary team evaluated wide local excision, including the possibility of partial penectomy, versus systemic targeted therapy. The patient ultimately underwent partial penile resection, resulting in marked improvement in pain and overall clinical status.

**Discussion:**

Penile PHE can mimic other spindle cell neoplasms, necessitating immunohistochemistry for definitive diagnosis. Surgery with negative margins remains the mainstay of treatment, though it may compromise function in genital sites. Targeted therapies directed at FOSB-related molecular pathways may benefit from unresectable or multifocal disease.

**Conclusion:**

There are only a limited number of cases reported worldwide of this rare neoplasm, and to our knowledge this represents the first reported case from Ecuador and the first from Latin America involving this anatomic site. It highlights the tumor’s diagnostic complexity, potential for aggressive local behavior, and the importance of multidisciplinary management and long-term surveillance.

## 1 Introduction

Pseudomyogenic hemangioendothelioma (PHE) is a rare vascular neoplasm of intermediate malignancy that predominantly affects the limbs, trunk, and head and neck regions in young adult males. Histologically, it is characterized by sheets of epithelioid to spindle-shaped cells with eosinophilic cytoplasm. Due to its morphological overlap with both benign and malignant soft tissue tumors, PHE often poses significant diagnostic challenges and is frequently misdiagnosed at initial presentation. Although it typically demonstrates multifocal growth within tissue planes and carries a low metastatic potential, local recurrence is a well-recognized concern (1, 2).

Genital involvement of PHE is exceedingly rare, with primary penile localization representing an extreme rarity; to date, only approximately 10 cases have been reported in the literature. This uncommon site introduces unique diagnostic difficulties, as lesions may clinically mimic benign conditions such as epidermal inclusion cysts, further delaying accurate recognition. The limited number of reported cases also restricts evidence-based guidance and contributes to ongoing debate regarding optimal management strategies (1, 3).

Here, we describe the case of a 29-year-old male with penile PHE, underscoring the substantial diagnostic and therapeutic challenges associated with this uncommon entity. To our knowledge, this is the first reported case of PHE in Ecuador and the first involving the genital region in Latin America.

## 2 Case presentation

A 29-year-old previously healthy male presented 5 years earlier with a painful, ulcerated lesion localized to the ventral aspect of the prepuce. The mass progressively enlarged, prompting consultation with an urologist. Circumcision was indicated and performed 1 month later. Histopathological examination at an external institution reported an ‘undifferentiated malignant neoplasm’; however, the original descriptive details were not available in the referral documentation, as only the final diagnostic label accompanied the specimen.

**Figure 1:**
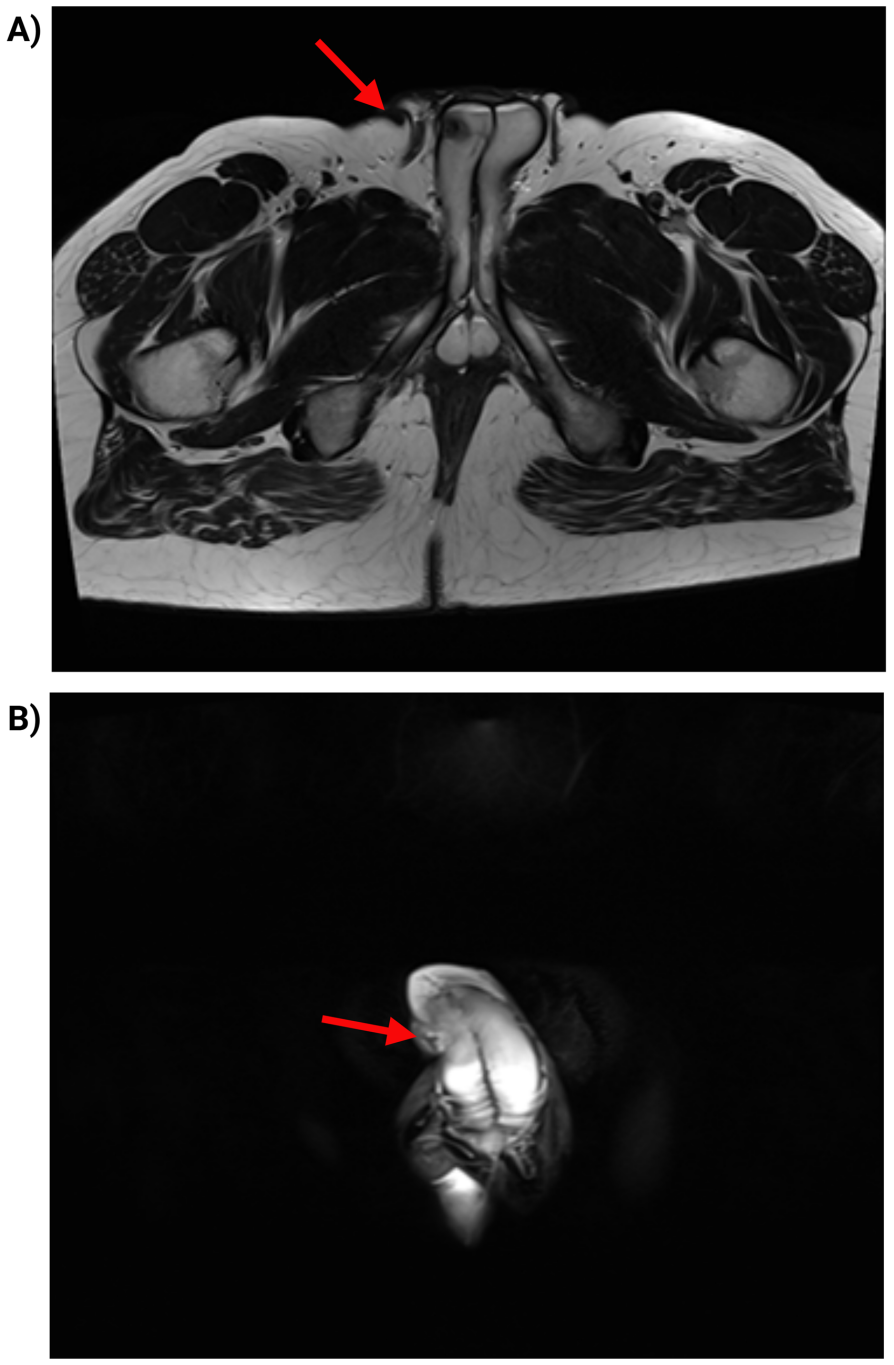
**(A)** T1-weighted MRI sequence demonstrating well-defined mass extending from the ventral penile shaft and infiltrating the corpus spongiosum, corpora cavernosa, and the glans penis, with a hypointense signal (arrowhead). **(B)** T2-weighted MRI sequence showing a nodular hyperintense lesion at the level of the penile shaft (arrow).

Despite surgical excision, the patient continued to experience pain, swelling, and erythema over the following 3 months. Magnetic resonance imaging (MRI) demonstrated a well-defined mass extending from the ventral penile shaft and infiltrating the corpus spongiosum, corpora cavernosa, and the glans penis (Figure 1).

**Figure 2:**
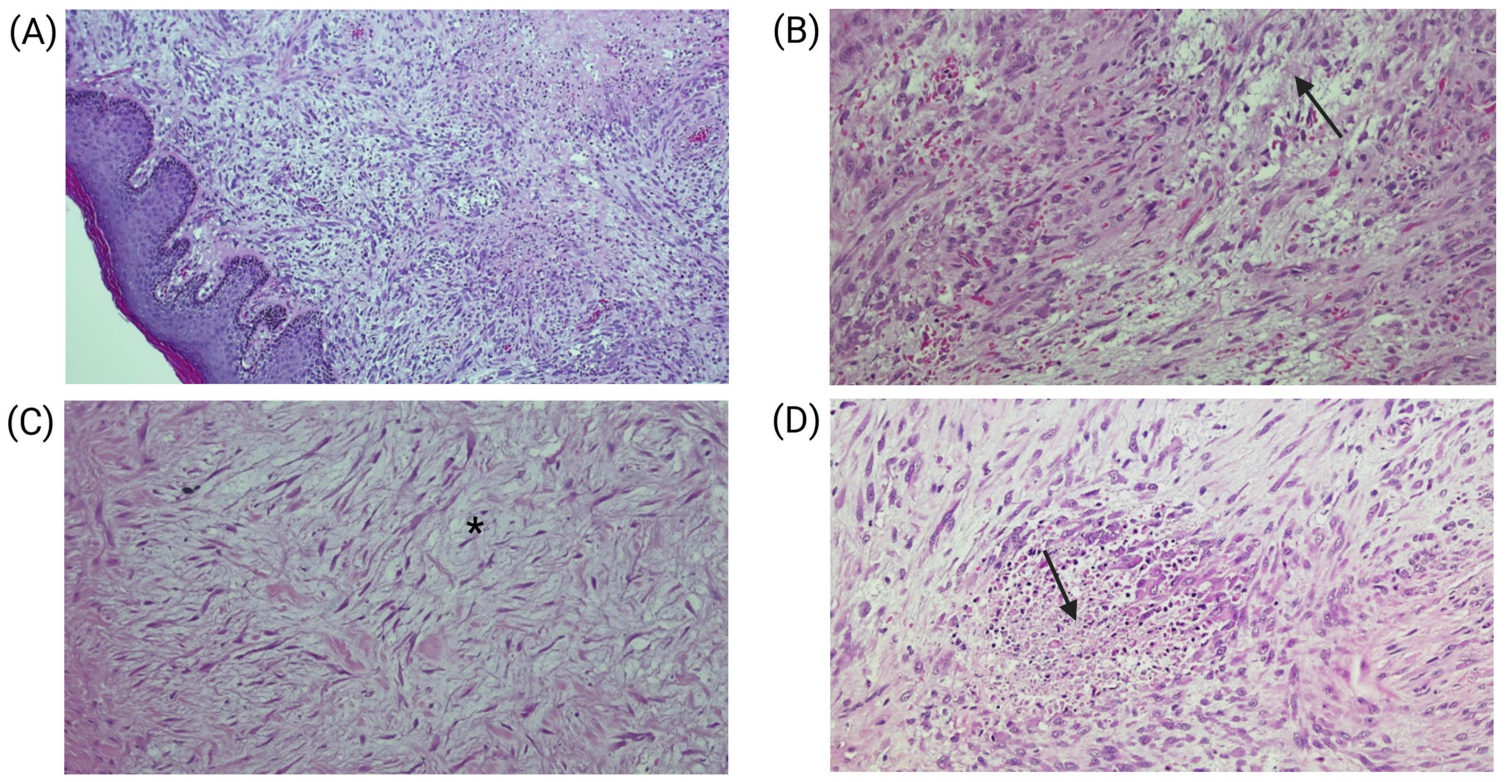
**(A)** Dermal infiltration of the lesión showing epithelioid cells arranged in nests, cords, and solid areas within a hyalinized to myxoid stroma (asterisk) (H&E 20×). **(B)** Pseudoangiomatous vascular spaces lacking true endothelial lining (arrowhead) (H&E 20×). **(C)** Mixoid stroma (H&E 20X). **(D)** Area of necrosis (arrow) (H&E 20×).

A subsequent histopathological review of the initial specimen, including detailed morphological evaluation and immunohistochemical analysis, confirmed the diagnosis of pseudomyogenic hemangioendothelioma. Histologically, the specimen showed foreskin tissue with ulcerated epidermis and an infiltrative dermal lesion composed of epithelioid cells arranged in nests, cords, and solid areas within a hyalinized to myxoid stroma (Figures 2A,C). Pseudoangiomatous vascular spaces lacking a true endothelial lining were observed (Figure 2B). The tumor cells exhibited abundant eosinophilic cytoplasm, vesicular nuclei with prominent nucleoli, and moderate nuclear atypia. The tumor demonstrated a high mitotic rate (7 mitoses per 10 high-power fields), with associated focal necrosis, microhemorrhages, and a moderate polymorphonuclear inflammatory infiltrate dispersed throughout the stroma (Figure 2D). The lesion extended into the subcutaneous tissue, encircled neural structures, and demonstrated positive surgical margins.

**Figure 3:**
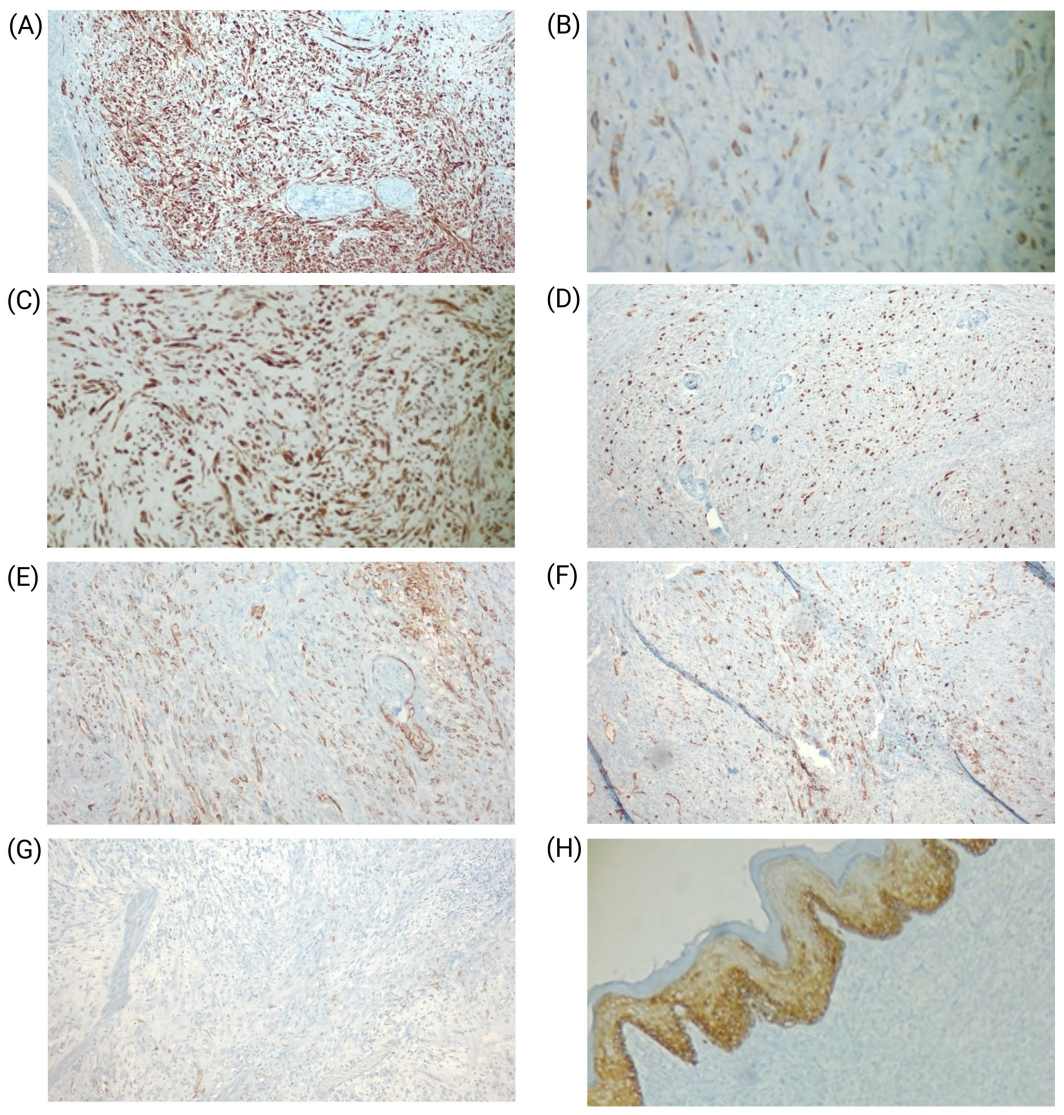
Immunohistochemical profile of the tumor. **(A)** Diffuse CK7 positivity in tumor cells (20×). **(B)** Strong cytoplasmic staining for AE1/AE3 (60×). **(C)** Vimentin expression in tumor cells (20×). **(D)** High proliferative index with Ki-67 labeling in approximately 55% of cells (20X). **(E)** CD31 positivity confirming endothelial differentiation (20×). **(F)** D2-40 expression in tumor cells (20×). **(G)** CD34 negativity (20×). **(H)** CK5/6 negativity in tumor cells (20×).

Immunohistochemically, the neoplastic cells demonstrated diffuse positivity for cytokeratins (CK7, AE1/AE3) and vimentin, supporting their epithelioid phenotype. Endothelial differentiation was confirmed by strong CD31 and D2-40 expression. The proliferation index, assessed by Ki-67, was markedly elevated at approximately 55%. In contrast, the tumor cells were negative for CD34 and CK5/6, thereby excluding alternative vascular and epithelial neoplasms (Figure 3). Although FOSB immunohistochemistry could not be performed due to limited local resources, we strongly recommend its inclusion in the diagnostic panel.

**Figure 4:**
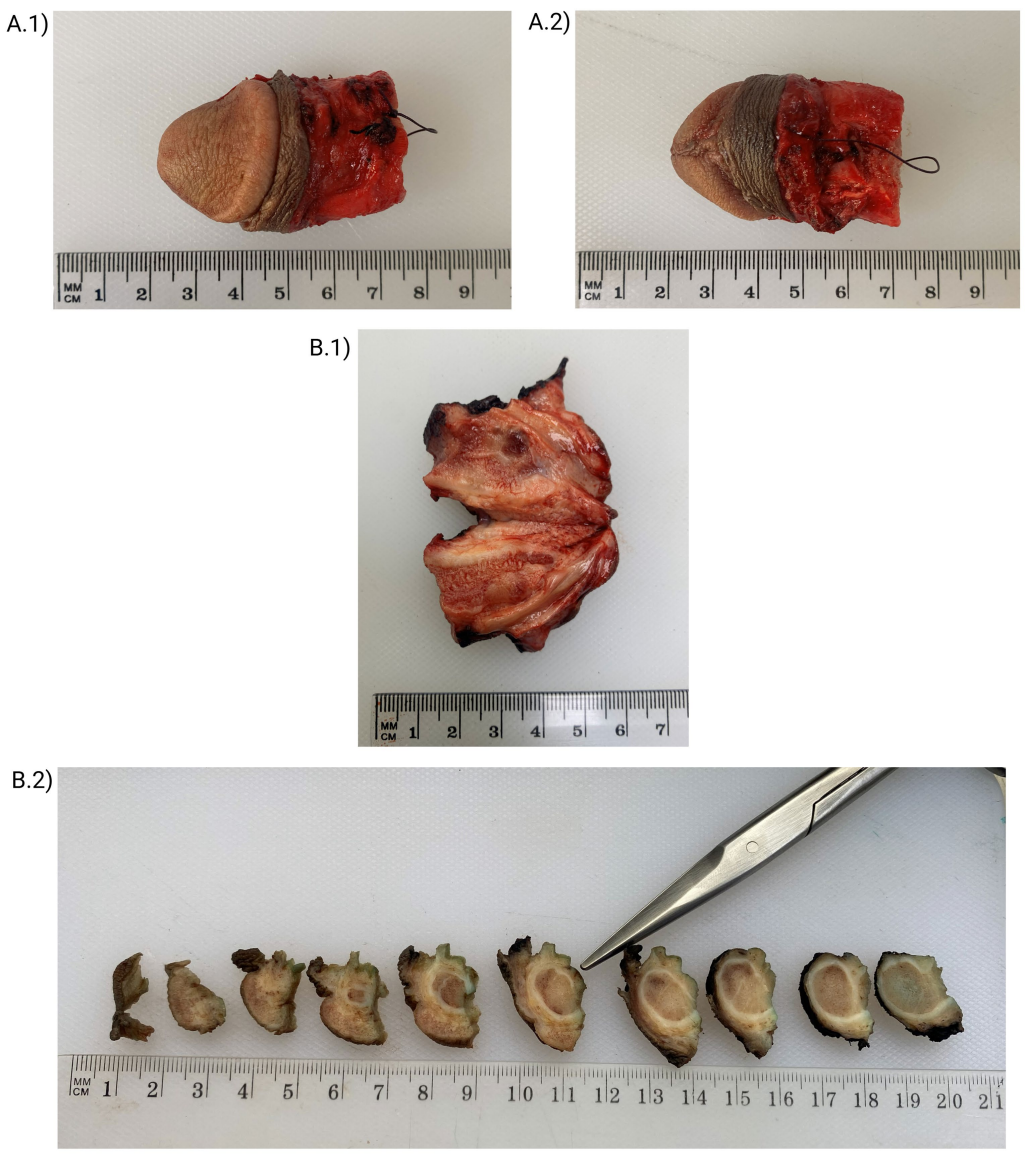
Grossing description. **(A.1,A.2)** Received in formalin is a partial penectomy specimen measuring 5.4 × 3.5 × 2.5 cm. The distal portion includes the glans penis, while the proximal resection margin appears irregular and hemorrhagic. **(B.1,B.2)** On serial sectioning, a firm, homogeneous, tan-violaceous lesion measuring 0.6 × 0.5 × 0.5 cm is identified within the corpus spongiosum. The lesion shows poorly defined borders and no gross evidence of necrosis or hemorrhage. The mass is located 2.2 cm from the proximal surgical margin. The remaining penile tissue appears unremarkable on gross examination. Representative sections are submitted for histopathologic evaluation, including the lesion and surgical margins.

**Figure 5:**
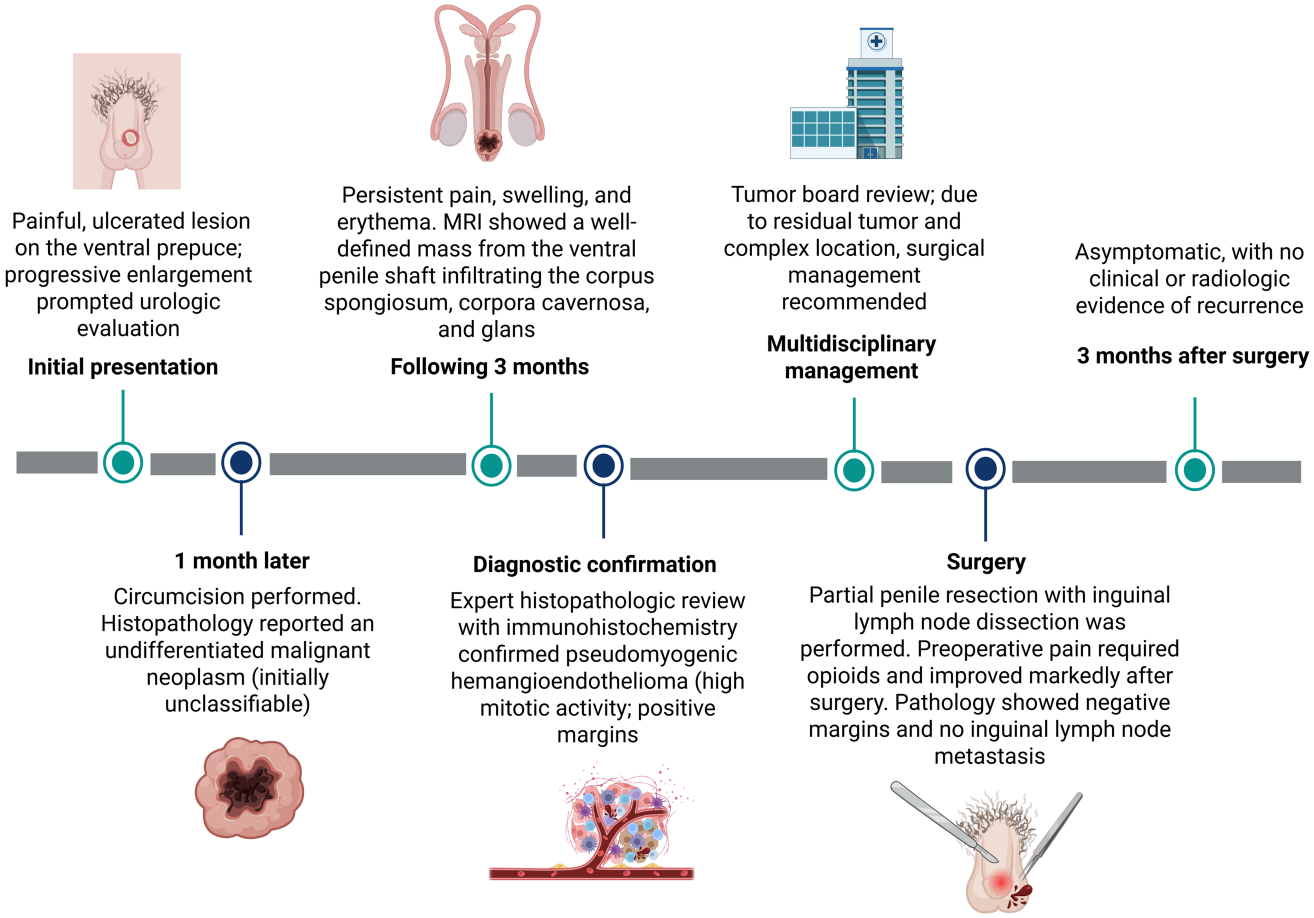
Timeline of the patient’s clinical course, diagnostic work-up, multidisciplinary decision-making, surgical management, and follow-up. Created with BioRender.com.

Given the complex location and the presence of residual tumor, a multidisciplinary evaluation was undertaken involving radiologists, surgical oncologists, and psycho-oncologists. The patient underwent surgical management with partial resection of the penis; grossing description of the specimen is describing (Figure 4). Prior to surgery, he experienced severe pain requiring opioid analgesia for symptom control. Following surgical intervention, there was significant improvement in pain and overall clinical status. At the most recent follow-up, 3 months after surgery, the patient remains asymptomatic with no clinical or radiologic evidence of tumor recurrence. The timeline of events for this case is summarized in Figure 5.

## 3 Discussion

PHE is a rare vascular neoplasm that is frequently misdiagnosed, particularly when it arises in unusual anatomical sites. In the present case, the tumor developed in the prepuce and penis, a location in which only a handful of cases have been reported to date (1, 4). At uncommon sites, PHE often exhibits deceptively bland histologic features and may mimic benign lesions or other malignancies. Indeed, up to half of reported PHE cases have been initially misdiagnosed (1).

Histopathologically, PHE typically demonstrates sheets of epithelioid and spindle-shaped cells with abundant eosinophilic cytoplasm and minimal atypia, but with little overt vascular differentiation (3). This renders differential diagnosis challenging, with key mimics including epithelioid sarcoma, epithelioid hemangioendothelioma, dermatofibrosarcoma protuberans, and rhabdomyosarcoma (5). Immunohistochemistry is therefore critical: PHE generally co-expresses cytokeratins and endothelial markers while lacking CD34, S100, and desmin. Nuclear FOSB expression serves as a sensitive surrogate marker for the characteristic SERPINE1–FOSB gene fusion, reflecting fusion-driven upregulation of the FOSB transcription factor; however, its diagnostic utility is limited by incomplete specificity as it may also be observed in epithelioid hemangioma, epithelioid sarcoma, nodular fasciitis, epithelioid angiosarcoma, and cellular benign fibrous histiocytoma, as well as in reactive endothelial cells and myofibroblasts in granulation or scar tissue (1, 6–8). In summary, the rarity of genital PHE and its nonspecific morphology necessitate a high index of suspicion and a comprehensive immunohistochemical panel to secure the diagnosis (3).

While our case demonstrated a high proliferative index (Ki-67 ≈ 55%) and an elevated mitotic rate, most published PHE series report lower proliferation indices and limited mitotic activity. In a recent clinicopathological study of 10 PHE cases, the Ki-67 index ranged from approximately 10–35%, and mitotic figures were described as rare or inconspicuous in most tumors (3). Similarly, other reports have described low mitotic activity (e.g., <5 mitoses per 50 high-power fields), consistent with the intermediate malignant behavior observed in most cases (1, 3, 9–11). Although the prognostic significance of Ki-67 and mitotic rate in PHE has not been systematically defined, these comparative data suggest that the elevated proliferative activity in our case may reflect a more locally aggressive phenotype. Nevertheless, interpretation should be cautious given the rarity of the disease and the lack of standardized reporting; larger series are needed to clarify prognostic value.

We summarized the main differential diagnoses of epithelioid neoplasms—particularly sarcomatoid carcinoma, epithelioid sarcoma, and epithelioid hemangioendothelioma—in Table 1 (12).

**Table 1:** Main histologic and immunohistochemical features distinguishing pseudomyogenic hemangioendothelioma (PHE) from its principal mimics.

Diagnosis	Typical sex/age	Growth pattern/architecture	Nodules with central necrosis	Intracytoplasmic vacuoles	Neutrophilic inflammatory infiltrate	Keratin (IHC)	CD31	CD34	INI 1
Sarcomatoid carcinoma	Male/57	Pleomorphic spindle/epithelioid cells; not classically cord-forming	−	Rare	May be present	Strong/diffuse +	−	−	Not usually performed
Epithelioid sarcoma	Male/ 23–43	Nodular growth, frequently with central necrosis	+++	Rare	Lymphocytic infiltrate common	Strong + (AE1/AE3, EMA)	− (usually)	Often + (variable)	−
Epithelioid hemangioendothelioma	Male/0–93	Vascular/cord-like growth in myxohyaline stroma	Variable	Common	May be present	Focal/variable	Variable (±)	Often +	Not usually performed
Pseudomyogenic hemangioendothelioma (PHE)	Male/30	Sheets, nests and cords of epithelioid cells; hyalinized–myxoid stroma	− to focal	Rare	Often marked (+++)	Variable: focal → diffuse +/++	Typically, + (strong)	Typically −	+++

Most PHEs arise in the distal extremities of young adult males (5). However, an increasing number of reports describe tumors in atypical locations. Penile involvement is exceptionally rare: prior to 2024, only five primary penile PHEs had been described. Youssef et al. recently reported five additional cases, highlighting that such lesions may clinically resemble indolent skin cysts (1). PHE has also been reported in the breast, oral cavity, chest wall, esophagus, and other trunk sites. A recent review of 189 cases found that ~74% occurred in the limbs (59% in the legs), with the remainder affecting the head and neck, trunk, or unusual sites such as the oral mucosa, vulva, and breast. Multifocality across tissue planes was common (71%). Infiltration into subcutaneous fat, muscle, bone, and nerve fibers is frequent (3). Similar to our case, which demonstrated vascular and perineural invasion, many PHEs show ill-defined infiltrative margins and perineural spread (9). Such features, along with the frequent presence of positive surgical margins, underscore the importance of wide excision and careful pathological assessment across all anatomical sites.

Surgery remains the cornerstone of PHE management. Wide local excision with negative margins is recommended whenever feasible. In the review by Yang et al., 80% of patients underwent surgical resection, with only a minority requiring adjuvant therapy. In our patient, partial penectomy with excision of the involved urethra and corpus spongiosum was necessary, reflecting the tumor’s aggressive local extension. Unfortunately, the diffuse and often multifocal nature of PHE means that even extensive resections may not be curative, and local recurrence is common (1).

Chemotherapy has no well-established standard role in the management of pseudomyogenic hemangioendothelioma and is generally reserved for unresectable, multifocal, or metastatic disease. Historically, cytotoxic regimens have been used sporadically with variable and often limited efficacy, contributing to the perception that PHE is relatively chemoresistant and that treatment-related toxicity may outweigh benefit. However, data on systemic therapy remain limited. In a recent multicenter study by Cai et al., 16 patients with PHE were analyzed, nearly half of whom had multifocal disease and a relatively high incidence of metastasis. Nine patients received chemotherapy, most commonly gemcitabine plus docetaxel, primarily with palliative intent. In this subgroup, encouraging short-term disease control was observed, with a 1-year progression-free survival rate of 80%, an overall response rate of 60%, and a disease control rate of 100%. Notably, treatment-related adverse events were manageable, with no grade 4 or 5 toxicities reported. Although these findings suggest that selected patients with advanced PHE may derive palliative benefit from gemcitabine-based chemotherapy, the small cohort size and retrospective nature of available studies preclude definitive conclusions, underscoring the need for further investigation and individualized treatment decisions (12, 13).

By contrast, targeted therapies have shown more promising activity. Several case reports describe meaningful responses to multitargeted tyrosine kinase inhibitors and mTOR inhibitors. For instance, pazopanib (a VEGFR/PDGFR inhibitor) led to clinical and radiologic improvement in a patient with multifocal PHE (14), while telatinib (a VEGFR1–4/PDGFR inhibitor) achieved complete remission in an adolescent with unresectable disease (15). Mechanistically, PHE’s FOSB fusion upregulates PDGFRA and VEGF signaling, making these agents rational therapeutic options (16). Similarly, mTOR inhibitors have demonstrated efficacy: sirolimus stabilized relapsed multifocal PHE in a pediatric patient (17), and everolimus induced complete metabolic regression of multifocal disease (18). Taken together, surgical resection remains the primary approach for local disease control, and systemic targeted therapies may be considered in selected cases of multifocal or refractory disease, although their role is not yet established.

Although PHE is classified as a low-grade malignant tumor with an overall favorable prognosis, multifocal recurrence remains a major clinical problem. In a review of 189 cases, 23% of patients experienced at least one recurrence, while 9% developed metastases—most commonly to soft tissue or lung. Death from PHE is uncommon, with only four fatal cases reported. Local recurrence, however, is frequent: in the series by Yang et al., four of 10 patients relapsed after initial surgery, although none developed metastases (3). Positive surgical margins, multifocal disease, and involvement of multiple planes appear to predict a higher risk of recurrence.

Our patient’s tumor exhibited extensive local spread, perineural invasion, and positive margins. Moreover, the unusual location and diagnostic delay may have contributed to its aggressive course. These factors highlight the importance of close follow-up. In published series, strict clinical surveillance—supplemented with imaging when appropriate—is recommended for several years, as recurrences can occur late (1). We summarized previously published patient demographics, clinical characteristics, pathological features, and treatments in Table 2.

**Table 2:** Summarize of previously published demographic, clinical, and pathological features of patients with penile pseudomyogenic hemangioendothelioma along with their respective treatments.

Author	Country	Age	Location	CD31	ERG	AE1/AE3	FOSB	Treatment	Time from onset to treatment	Outcome/remarks
Current case	Ecuador	29	Ventral prepuce, invading glans, corpus spongiosum & corpora cavernosa	+	+	+	ND	Circumcision initially; Then Surgical excision	~5 years from first symptoms until surgery	Follow-up surgery showed no evidence of disease after 3 months
Youssef et al. (1)	United States of America	47	Glans penis	+	+	+	+	mTOR inhibitor	Two months after the initial diagnosis	Follow-up showed no evidence of disease after 2 months of treatment.
Youssef et al. (1)	United States of America	53	Penis	+	+	+	ND	Surgical excision	Not specified	FOSB not done
Youssef et al. (1)	United States of America	28	Glans penis	+	+	+	+	Surgical excision	Not specified	Youngest in series
Youssef et al. (1)	United States of America	56	Penis	+	+	+	+	Surgical excision	Not specified	Older patient
Youssef et al. (1)	United States of America	51	Glans penis	+	+	+	+	Surgical excision	Not specified	Complete marker profile
Hornik et al. (21)	United States of America	18	Penis (with scrotal/thigh involvement)	NA	NA	NA	NA	Surgical excision	Not specified	Metastasis to inguinal lymph node
Hornik et al. (21)	United States of America	27	Penis	Equivocal	ND	+	ND	Surgical excision	Not specified	Recurrence in 4 months, no mets
Zhou et al. (10)	China	37	Glans penis	Weak/patchy +	+	+	Rearrangement +	Surgical excision	Not specified	FOSB rearrangement confirmed
Ide et al. (11)	Japan	43	Penis	+	+	+	+	Surgical excision	6 months after diagnosis	The patient is disease-free 9 months after the re-excision.
Zhou et al. (10)	China	30	Glans penis	NA	NA	NA	NA	Not specified	Not specified	Not specified

Recent advances in molecular pathology have clarified the genetic landscape of PHE, with recurrent FOSB gene fusions recognized as a defining molecular event. These rearrangements, including SERPINE1:FOSB and ACTB:FOSB fusions, involve the N-terminal region of the protein with preservation of the DNA-binding basic region, leucine zipper, and transactivation domains, leading to FOSB upregulation through promoter swap mechanisms. (6, 19, 20).

Functionally, FOSB encodes a member of the Fos family of transcription factors, which heterodimerize with Jun proteins to form the activator protein-1 (AP-1) complex. AP-1 binds promoter and enhancer elements and regulates transcriptional programs involved in cell proliferation, survival, angiogenesis, and invasion. In PHE, fusion-driven promoter swapping leads to aberrant FOSB overexpression, which is thought to dysregulate AP-1–mediated gene expression and contribute to tumorigenesis, including upregulation of angiogenic and growth signaling pathways (6, 8).

This report has several limitations. Molecular confirmation of FOSB gene rearrangement was not performed, which represents a diagnostic limitation; however, the diagnosis was supported by characteristic histomorphologic features and a highly specific immunophenotypic profile. In addition, the duration of follow-up is currently limited, precluding definitive conclusions regarding long-term disease control or recurrence. Ongoing clinical and radiologic surveillance is therefore essential, particularly given the known propensity of PHE for late local recurrence. Given the inherent limitations of a single case report, these observations are descriptive and intended to highlight diagnostic and management considerations rather than to support generalizable conclusions.

Finally, as a rare disease, pseudomyogenic hemangioendothelioma would benefit from the development of standardized diagnostic criteria and the establishment of multi-institutional registries and collaborative clinical trials to improve diagnostic consistency, refine prognostic markers, and optimize treatment strategies. In the meantime, insights derived from case series and well-documented reports will continue to inform clinical practice, particularly in guiding risk stratification, tailoring adjuvant therapies for high-risk patients, and incorporating targeted agents when conventional management proves inadequate.

## 4 Conclusion

This exceptionally rare case of penile PHE underscores the significant diagnostic and therapeutic challenges posed by this tumor in anatomically and functionally sensitive locations. Beyond its rarity, this report highlights the clinical consequences of this neoplasm, emphasizing the pivotal role of comprehensive immunohistochemical analysis in establishing an accurate diagnosis in tumors with non-specific morphology that may mimic benign lesions or other malignancies at unusual sites. This case contributes to the limited global literature on genital PHE and represents, to our knowledge, the first documented case in Ecuador and the first involving the genital region in Latin America. Furthermore, it illustrates the importance of multidisciplinary decision-making, including consideration of emerging targeted therapies for recurrent, multifocal, or unresectable disease, and underscores the necessity of long-term clinical and radiologic surveillance given the substantial risk of local recurrence inherent to PHE.

## Data Availability

The original contributions presented in the study are included in the article/supplementary material, further inquiries can be directed to the corresponding author.

## References

[1] Youssef R Davis JL Anderson WJ Acosta AM. Pseudomyogenic hemangioendothelioma presenting as a penile lesion. Virchows Arch. (2024) 485:1157–60. doi: 10.1007/s00428-024-03944-z, 39432093

[2] Gersmann A Haller F Behnert N Richter A Stöhr R Hartmann A . Primary pseudomyogenic haemangioendothelioma of the testis with a novel POTEI::FOSB gene fusion. Histopathology. (2022) 81:411–4. doi: 10.1111/his.14697, 35582841

[3] Yang N Huang Y Yang P Yan W Zhang S Li N . Clinicopathological study of pseudomyogenic hemangioendothelioma. Diagn Pathol. (2023) 18:1–7. doi: 10.1186/s13000-023-01309-9 36803395 PMC9940391

[4] Ge Y Lin X Zhang F Xu F Luo L Huang W . A rare case of pseudomyogenic hemangioendothelioma (PHE)/epithelioid sarcoma-like hemangioendothelioma (ES-H) of the breast first misdiagnosed as metaplastic carcinoma by FNAB and review of the literature. Diagn Pathol. (2019) 14:1–7. doi: 10.1186/s13000-019-0857-6 31311568 PMC6635997

[5] Choi ME Lim DJ Chang SE Lee MW Choi JH Lee WJ. A case of pseudomyogenic hemangioendothelioma of the lower extremity. Ann Dermatol. (2020) 32:426. doi: 10.5021/ad.2020.32.5.426, 33911779 PMC7992577

[6] Cordier F Creytens D. New kids on the block: FOS and FOSB gene. J Clin Pathol. (2023) 76:721–6. doi: 10.1136/jcp-2023-208931, 37553246

[7] Tsui KY Maclean F Moir D Cheah A Bonar F Tabot J . Immunohistochemistry for FOSB and FOS is a useful ancillary tool in the diagnosis of epithelioid hemangioma but there are pitfalls in interpretation including expression in other vascular lesions. Int J Surg Pathol. (2023) 31:280–8. doi: 10.1177/10668969221101867, 35635207

[8] Hung YP Fletcher CDM Hornick JL. FOSB is a useful diagnostic marker for pseudomyogenic hemangioendothelioma. Am J Surg Pathol. (2017) 41:596–606. doi: 10.1097/PAS.0000000000000795, 28009608

[9] Otani S Nakayama R Sekita T Hirozane T Asano N Nishimoto K . Pseudomyogenic hemangioendothelioma of bone treated with denosumab: a case report. BMC Cancer. (2019) 19:872. doi: 10.1186/s12885-019-6072-8, 31481040 PMC6724307

[10] Zhou J Yu D Chen X Wang J Cai S. Pseudomyogenic hemangioendothelioma in the external genitalia. J Dtsch Dermatol Ges. (2024) 22:700–3. doi: 10.1111/ddg.15353, 38581344

[11] Ide YH Tsukamoto Y Ito T Watanabe T Nakagawa N Haneda T . Penile pseudomyogenic hemangioendothelioma/epithelioid sarcoma-like hemangioendothelioma with a novel pattern of SERPINE1-FOSB fusion detected by RT-PCR – report of a case. Pathol Res Pract. (2015) 211:415–20. doi: 10.1016/j.prp.2015.02.003, 25749627

[12] Cai Q Xu B Zhang P Ma J Liang J Le L . The efficacy and safety of gemcitabine and docetaxel in pseudomyogenic hemangioendothelioma: a multi-center experience. BMC Cancer. (2025) 25:1030. doi: 10.1186/s12885-025-14376-6, 40597841 PMC12210678

[13] Joseph J Wang WL Patnana M Ramesh N Benjamin R Patel S . Cytotoxic and targeted therapy for treatment of pseudomyogenic hemangioendothelioma. Clin Sarcoma Res. (2015) 5:22. doi: 10.1186/s13569-015-0037-8 26500758 PMC4615364

[14] Alhanash A Aseafan M Atallah J. Pazopanib as treatment option for pseudomyogenic hemangioendothelioma: a case report. Cureus. (2022) 14:e25250. doi: 10.7759/cureus.25250, 35755544 PMC9216676

[15] Van IJzendoorn DGP Sleijfer S Gelderblom H Eskens FALM Van Leenders GJLH Szuhai K . Telatinib is an effective targeted therapy for pseudomyogenic hemangioendothelioma. Clin Cancer Res. (2018) 24:2678–87. doi: 10.1158/1078-0432.ccr-17-3512, 29511030

[16] Sun YF Wang J. Primary pseudomyogenic hemangioendothelioma of the vulva: a rare location for a rare entity. Diagn Pathol. (2019) 14:66. doi: 10.1186/s13000-019-0846-9, 31238962 PMC6593540

[17] Gabor KM Sapi Z Tiszlavicz LG Fige A Bereczki C Bartyik K. Sirolimus therapy in the treatment of pseudomyogenic hemangioendothelioma. Pediatr Blood Cancer. (2018) 65:e26781. doi: 10.1002/pbc.26781, 28843050

[18] Ozeki M Nozawa A Kanda K Hori T Nagano A Shimada A . Everolimus for treatment of pseudomyogenic hemangioendothelioma. J Pediatr Hematol Oncol. (2017) 39:e328–31. doi: 10.1097/MPH.0000000000000778, 28121744

[19] Panagopoulos I Lobmaier I Gorunova L Heim S. Fusion of the genes WWTR1 and FOSB in pseudomyogenic hemangioendothelioma. Cancer Genomics Proteomics. (2019) 16:293–8. doi: 10.21873/cgp.20134, 31243110 PMC6609259

[20] Agaram NP Zhang L Cotzia P Antonescu CR. Expanding the spectrum of genetic alterations in pseudomyogenic hemangioendothelioma with recurrent novel ACTB-FOSB gene fusions. Am J Surg Pathol. (2018) 42:1653–61. doi: 10.1097/PAS.0000000000001147, 30256258 PMC6608746

[21] Hornick JL Fletcher CDM. Pseudomyogenic hemangioendothelioma: a distinctive, often multicentric tumor with indolent behavior. Am J Surg Pathol. (2011) 35:190–201. doi: 10.1097/PAS.0b013e3181ff0901, 21263239

